# Protegrin 1 Enhances Innate Cellular Defense via the Insulin-Like Growth Factor 1 Receptor Pathway

**DOI:** 10.3389/fcimb.2018.00331

**Published:** 2018-09-28

**Authors:** Jenna Penney, Julang Li

**Affiliations:** ^1^Department of Life Science and Engineering, Foshan University, Foshan Shi, China; ^2^Department of Animal Bioscience, University of Guelph, Guelph, ON, Canada

**Keywords:** Protegrin-1, antimicrobial peptides, immune modulation, cell migration, inflamation

## Abstract

Antimicrobial peptides (AMPs) represent a promising area of research to help combat the ever-growing problem of antibiotic resistance. Protegrin-1 is an AMP from the cathelicidin family. It is produced naturally in pigs and its mature form (mPG-1) has potent bactericidal properties and a unique β-hairpin structure that separates it from most AMPs found in mice and humans. While the antibacterial properties of protegrin-1 are well established, the role it plays in immune modulation has yet to be investigated, and our current study sought to explore this alternate role and potential mechanism behind. We found that mPG-1 stimulated intestinal cell migration, this is accompanied with altered expression of genes associated with cell migration, in addition to increased expression of pro-inflammatory cytokines and immune-related factors. Further study suggested that mPG-1 activates insulin-like growth factor 1 receptor (IGF1R) and through this receptor it modulates immune activity as well as cell migration. Our study revealed a novel function of mPG-1, and its associated pathway, suggesting therapeutic potential of the antimicrobial peptide for infection and/or immune disorders, particularly ones affecting the gastrointestinal tract such as inflammatory bowel syndrome.

## Introduction

Resistance to commonly used antibiotics is a growing problem in both human and veterinary medicine, which makes developing alternatives an important area of research (Munita and Arias, 2016). Antimicrobial peptides (AMPs) are a diverse class of naturally occurring molecules that are produced by all multicellular organisms as a first line of defense. These proteins have broad activity to directly kill bacteria, yeasts, fungi, and enveloped viruses (Scott et al., 1999). The mode of action of AMPs involves direct electrostatic interaction with negatively charged microbial cell membranes, followed by membrane permeabilization and physical disruption (Hancock and Lehrer, 1998; Hancock and Scott, 2000). In contrast, conventional antibiotics commonly work through targeting specific enzymes to inhibit important basal metabolic processes in bacteria, which in turn kills the bacteria (Munita and Arias, 2016). The conventional mechanism of action is prone to allowing for bacteria to develop a resistance to commonly used antibiotics. This can occur through single nucleotide polymorphisms (SNPs) and can render bacteria partially or completely resistant to antibiotics that tend to be overused. As most AMPs kill bacteria through a physical means, (based on ionic and hydrophobic interactions) and specific receptors/protein targets are not commonly involved, bacterial resistance is more difficult to develop (Hancock and Scott, 2000). The selective bactericidal activity of AMPs on microbial cells as opposed to mammalian cells is suggested to be caused by the high content of anionic lipids on the surface of the bacterial membrane, the high electrical-potential gradient across this membrane, and the absence of cholesterol in this membrane (Hancock and Lehrer, 1998).

Insects and plants primarily deploy AMPs as a measure to protect against potential pathogens (Wang, 2014). In higher eukaryotic organisms, AMPs can have additional immunomodulatory activities and are often referred to as host defense peptides. These activities vary depending on the AMP and the effects can be diverse, including a variety of cytokine and pro-inflammatory effects that are relevant to normal immune system homeostasis (Scott and Hancock, 2000). Altered expression of AMPs has been shown to be involved in various autoimmune diseases, highlighting the importance of understanding these molecules not only for their antimicrobial activities but also the modulating effects they exert on the immune response (Kahlenberg and Kaplan, 2013; Marcinkiewicz and Majewski, 2016).

Cathelicidin is a class of AMPs endogenously expressed in epithelial and immune cells and the expression has been shown to increase drastically during infection and inflammation (Lee et al., 2011). Various cathelicidins present in different mammals have been reported to exert chemotactic activity on neutrophils, monocytes, dendritic cells and T-cells. Furthermore, cathelicidins have been shown to enhance angiogenesis and wound healing (Dommisch et al., 2015).

Protegrins (PGs) are a family of cathelicidin peptides that are produced by neutrophils in pigs, there are five family members, PG-1, 2, 3, 4, and 5. They exist initially in the pro-form (pro-PG), with two domains consisting of an evolutionally conserved pro region called “cathelin domain”, and a downstream C-terminal domain containing the active antimicrobial peptide, mature PG (mPG-1) (Storici and Zanetti, 1993). Protegrin 1 (PG-1) is the most abundant and well characterized member of the protegrins family produced in pigs. PG-1 has attracted much attention due to its antimicrobial potency and its relative robustness *in vivo* (Scott and Hancock, 2000; Lee et al., 2005, 2011; Khandelia and Kaznessis, 2007; Cheung et al., 2008; Capone et al., 2010; Jang et al., 2011; Wuerth and Hancock, 2011), however its role in immune modulation is unclear.

Recent studies suggest that the function of the cathelicidin family is not limited to the killing of bacteria. These small peptides may exert broader functions as an integral part of the innate immune system, exerting either immunostimulating or immune-modulating effects (Braff et al., 2005; van Wetering et al., 2005; Hancock and Sahl, 2006; Wuerth and Hancock, 2011). Although protegrin is from the cathelicidin family of AMPs, it has a β-hairpin structure, in contrast to the α-helical peptides of mouse and human cathelicidin (Khandelia and Kaznessis, 2007). Further understanding of the role and mechanism of PG-1 action may provide insights into the physiology of the AMP, which may assist in defining the therapeutic potential of this important AMP in various immune related disorders. The objective of the present study was to examine the immune-mediating and potential tissue repair function of mPG-1, and to identify the pathway(s) that mPG-1 may exert its effects through.

## Materials and methods

### Cell culture

All cell types were grown in monolayer culture in Dulbecco's modified Eagle's medium (high glucose) supplemented with 10% (vol/vol) fetal bovine serum (Invitrogen), 100 IU/ml penicillin, and 100 g/ml streptomycin. All cultures were maintained in a 5% CO2 humidified atmosphere at 37°C and passaged every 2 to 3 days. Cells were plated 24 h prior to transfection and allowed to grow to 60% confluence prior to transfection. Cells were transfected by polyjet transfection reagent (SignaGen Laboratories) as per the manufacturer's instruction^1^.

### Transwell (cell migration) assay

A cell line established from intestinal porcine enterocytes isolated from the jejunum of a neonatal unsuckled piglet (IPEC-J2 cells, DSMZ^*^), 8-micron pore sized cell culture transwell inserts were used (Millipore Inc, Temecula, CA). A total of 1 × 10^5^ cells were plated in the upper inserts and the lower chamber contained serum-free DMEM F-12 in the absence and presence of 1, 4.5, or 9 μM (approximately equivalent to 3, 10, or 20 μg/mL) of synthetically synthesized mature PG-1. After incubation for 16 h, the cells were fixed with 4% (w/v) paraformaldehyde. Cells that did not migrate into the membrane were gently scraped off the upper surface of the transwell with a cotton swab. Migration was quantified by cell enumeration through Hoechst 33342 staining of cell nuclei (Life Technologies).

### RNA analysis and qRT-PCR

Total RNA was isolated from IPEC-J2 cells after the cells were serum starved from 24 h, using a Norgen total RNA isolation kit (Thorold, ON, Canada). cDNA was synthesized from total RNA using All in one 5X reverse transcriptase (AMB, Richmond, BC, ON). Transcript levels were measured by quantitative RT-PCR (qRT-PCR) using PerfeCTa SYBR green Supermix with 6-carboxy-X-rhodamine (ROX) (Quanta Biosciences, Inc., Gaithersburg, MD) and primers against pig genes. Samples were run on a BioRad CFX Connect Real-time system and subjected to standard curve analysis, and arbitrary values were represented, adjusting for primer efficiencies. Primer sequences are provided in Table 1.

**Table 1:** Primer sequence used for qRT-PCR.

**Gene**	**Forward primer**	**Reverse primer**
CCL2	TCAGGCATGGAGGTAGAACC	TAACAGCACTCCCTTCCCTCT
COX2	ATGATCTACCCGCCTCACAC	GCAGCTCTGGGTCAAACTTC
EGFR	GCCTTAGCCGTCTTATCCAA	TGGGCACAGATGACTTTGGT
IRF7	GGACTTGACCATCATGTACAAAGG	AGCTTCTCTGTGTAGTGGAGCTG
MCL1	GCCTCCAGAGAAACGCAGTA	TTTCCCGTAGCCAAGAGACG
MUC-1	CTCTGCTCAGCCTGGGTCT	GCTCATAGGATGGTAGGCA
ICAM	TGGCTGGGCATGTGCTATAC	GTCATTGTCCAGAGACCCCA
NFKB (subunit 1)	GAAGGACCTCTAGAAGGCAAAA	GCTTTGGTTTATGCGGTGTT
ECAD	CAGTGCCAACTGGACCATCG	CCCAGGATGGCAGGAACTTG
GAPDH^*^	AGCAATGCCTCCTGTACCAC	AAGCAGGGATGATGTTCTGG
YWH^*^	TGATGATAAGAAAGGGATTGTGG	GTTCAGCAATGGCTTCATCA

* reference genes used for normalization.

### Dual luciferase assay

HEK293 cell cultures were grown to approximately 70% confluence prior to transfection using polyjet (SignaGen Laboratories) using manufacturer's instructions. The cells were co-transfected with the plasmids from the Pathdetect trans-reporting system following manufacturers instructions (Agilent Technologies, Santa Clara, CA, USA). At 16–18 h post-transfection, the medium was replaced to and serum was removed for 24 h prior to collection. The cells were harvested, and dual luciferase assays were carried out according to the manufacturer's instruction (Promega). Reporter activity was calculated as relative luciferase activity (firefly luciferase/ *Renilla* luciferase) to correct for transfection efficiency.

### Assessing phosphorylation status of MAPK pathway

For the detection of the phosphorylation status of MAPKs and other serine/threonine kinases, the Human Phospho-MAPK Array kit (R&D Systems) was used according to the manufacturer's protocol, and 300 μg of protein lysate was used for each array. HEK293 cells were grown to 60% confluency, serum starved for 24 h followed by treatment with mPG (9μM) for 15 min. Lysate was collected using the lysis buffer provided with the kit and the protein level was assessed using a BCA assay (Pierce Biotechnology, USA). Equal amount of protein (300 μg) were added to each array and the manufacturer's instructions were followed. The pixel density in each spot of the array was determined by ImageJ software relative to control spots.

### Apoptosis and necrosis assay

Apoptosis and Necrosis was measured using the Real-time Glo Annexic V Apoptosis and Necrosis assay (Promega), assay was performed as directed in the protocol provided. Briefly, IPEC-J2 cells were plated in a 96-well plate at a density of approximately 5,000 cells/well. Sixteen hours after plating the cells were treated with the indicated treatment, the detection reagent was added and read on a plate reader at each indicated time point. Relative apoptosis was calculated as a proportion of the control wells.

### Alamar blue assay

Cell viability was measured using the AlamarBlue cell Viability reagent (ThermoFisher) and the instructions given in the protocol were followed. Briefly, IPEC-J2 cells were plated in a 96 well plate at a density of approximately 5,000 cells/well. After 24 h serum was removed and the cells were treated, 24 h after treatment the alamar blue reagent was added and measurements were taken at 1, 2, 4, and 24 h. Data were consistent at each measurement so only the 2 h time point is presented^2^.

### Statistical analysis

All the assays were independently repeated at least four times, and results are shown with standard error. Statistical analysis was done using a one-way ANOVA with *post hoc* tukey tests.

## Results

**Figure 1:**
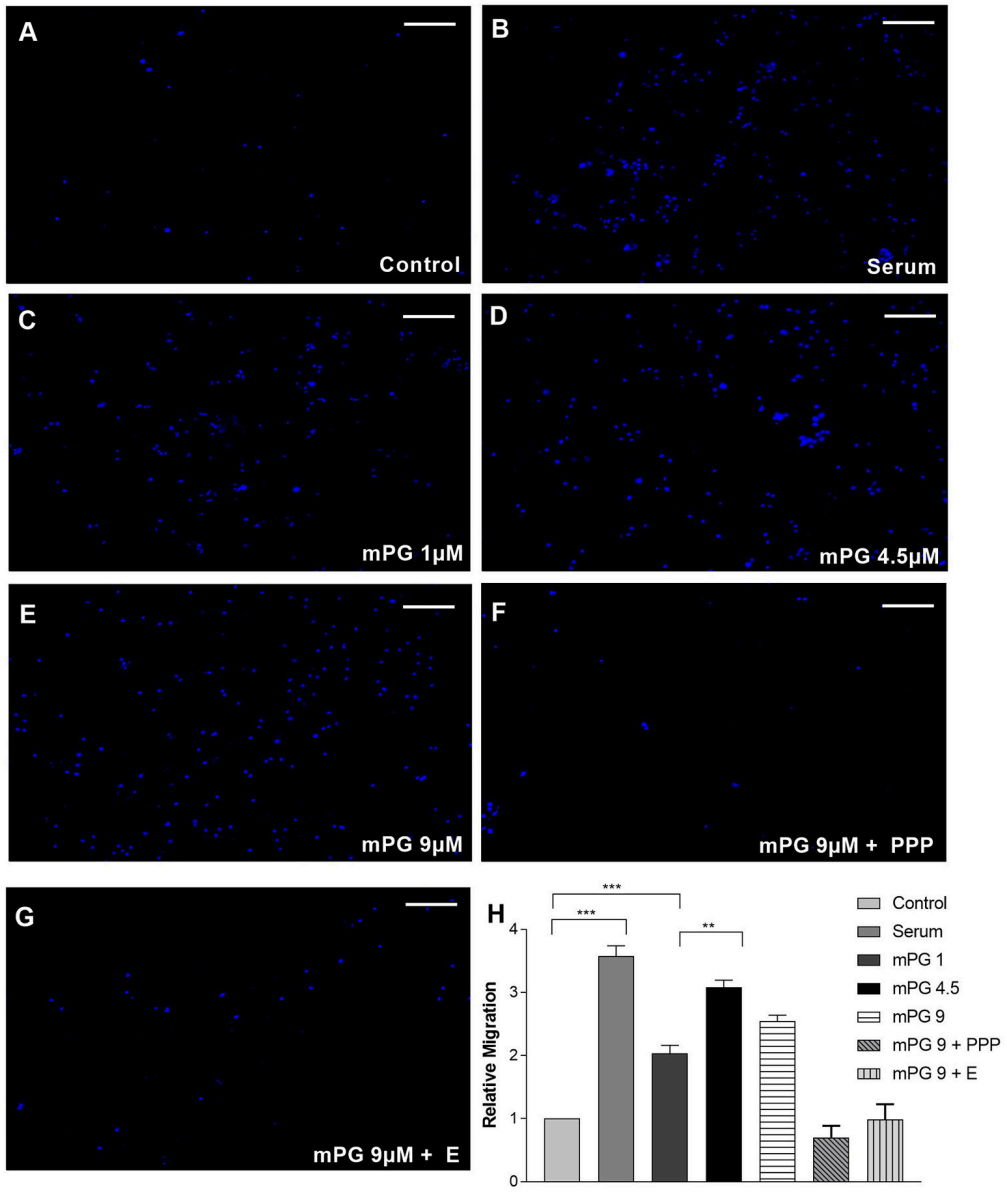
mPG-1 enhanced cell migration. **(A–G)** Representative images of transwell migrated IPEC-J2 cells stained with Hoechst 33342. **(H)** Quantification of cell migration from transwell migration assay. Bars represent the mean ± SEM of three experiments. One-way ANOVA with a *post hoc* Tukey test was used for analysis, **p < 0.01 and ***p < 0.001. Scale bar represents 300μm.

Cellular migration is an important process when it comes to wound healing in all tissues and these repair mechanisms help to keep pathogens from spreading throughout the body (Eming et al., 2014). This is of particular importance in the intestine where exposure to many pathogens is quite common (Cunliffe and Mahida, 2004). Our previous study has suggested that mPG1 promotes migration of intestinal porcine enterocytes (IPEC-J2 cells; unpublished data) we sought to first validate this finding. As shown in Figure 1, in the presence of various concentrations of mPG-1, cell migration was significantly increased compared to control. The effect appears to dose dependent, with the optimal concentration at 4.5 μM (*p* < 0.01). These results confirmed the role of mPG-1 in intestine cell migration. In the presence of the inhibitors for insulin like growth factor 1 receptor (IGF1R) and epidermal growth factor receptor (EGFR), Picropodophyllin (PPP) and erlotinib respectively, the enhancement of cell migration seen with mPG-1 treatment was reversed.

Cytotoxicity and apoptosis are elements that are important to investigate when using any treatments on new cells types. To assess the level of apoptosis and necrosis we performed a real-time assay, and found that while mPG-1 treatment slightly increased apoptosis in the first hour of treatment, in all other time points there was no significant difference from control. This was consistent in all mPG-1 concentrations used (4.5–18 μM) (Supplementary Figure 1A). To assess cytotoxicity, we performed an alamar blue assay and found that at 18 μM mPG-1 treatment the cell viability was significantly decreased compared to control, however this difference was not seen with treatment at 9 or 4.5 μM (Supplementary Figure 1B).

**Figure 2:**
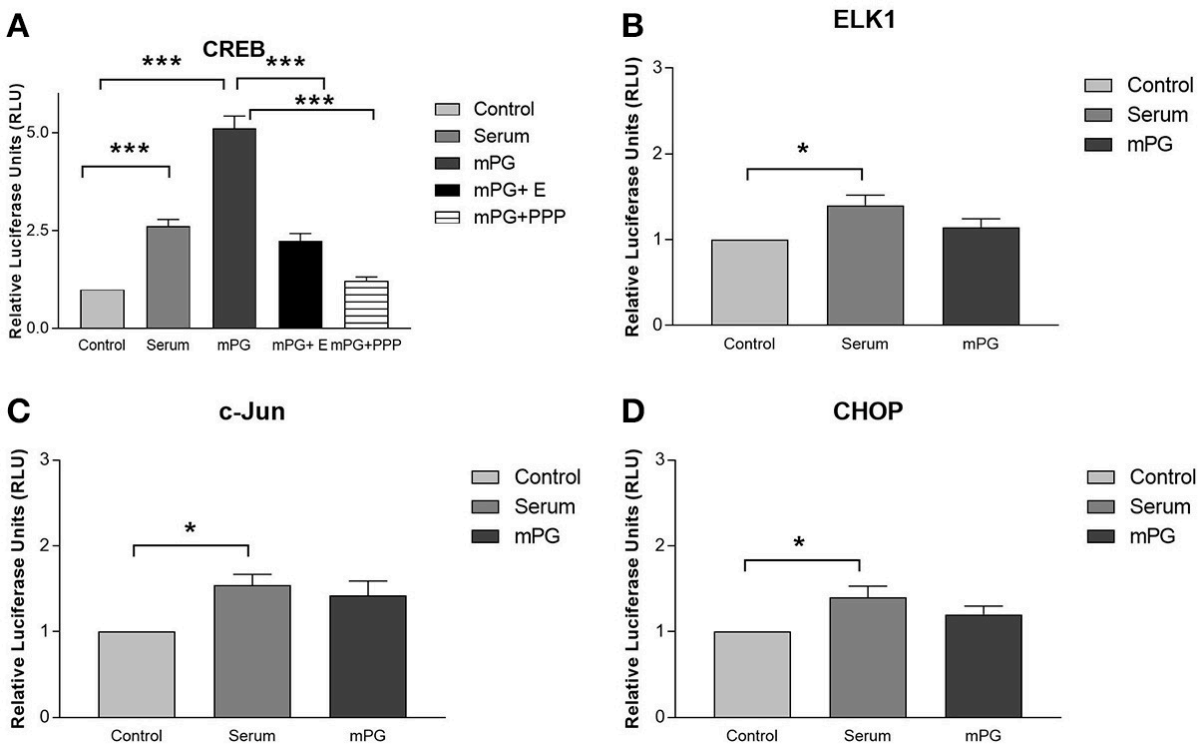
mPG-1 activates CREB. HEK293 cells were co-transfected with a plasmid, pFR-Luc- a luciferase reporter plasmid, containing a synthetic promoter with five tandem repeats of the GAL4 binding site, and pFA2-CREB**(A)**, pFA2-Elk1 **(B)**, pFA2-C-Jun **(C)** or pFA2-CHOP **(D)** that expresses the activation domain of each transcription factor fused to the GAL4 DNA binding domain. Sixteen to Eighteen hours after transfection the cells were serum starved and treated with 9 μM mPG-1 or 10% FBS for 16 hours. Luciferase activity, which is indicative of transcription factor dependent activation, is expressed as relative light units compared to control. The data was obtained in triplicate and normalized for transfection efficiency using a constitutively active Renilla reporter plasmid included in all transfections. Bars represent the mean ± SEM of five independent experiments for all experimental groups. One-way ANOVA with a *post hoc* Tukey test was used for analysis, *p < 0.05, ***p < 0.001. E, erlotinib; EGFR inhibitor; PPP: picropodophyllin IGF1R inhibitor.

EGFR and IGF1R pathways are two important pathways that are involved in cell migration and immune modulation (Smith, 2010; Schanzer et al., 2016). Two of the secondary pathways activated through these receptors are the ERK and AKT pathways which can result in the activation of integral transcription factors including c-Jun, Elk1, CREB and potentially, through secondary signaling, CHOP (Li et al., 2009; Christopoulos et al., 2015). To examine the influence of mPG-1 on these pathways, we used a well-established luciferase based path-detect system (agilent technologies) to monitor activation of these transcription factors. This system is an intricate reporter assay that allows for quantifying the activity of the specific transcription factor utilizing the yeast based Gal4 DNA binding domain. As expected, the inclusion of a constitutively active MEK expression plasmid in the co-transfection cocktail resulted in a significant increase of activation for each transcription factor (data not shown). As shown in Figure 2, mPG-1 treatment resulted in an increase of CREB activity, while no effect was observed when assessing the activity of c-Jun, CHOP or ELK-1. Furthermore, CREB activation by mPG-1 was completely blocked with the pre-treatment of Picropodophyllin (PPP), a potent and specific IGF1R inhibitor (Vasilcanu et al., 2006) (Figure 2A). On the other hand, an inhibitor of EGFR, erlotinib, suppressed the CREB activity, but not completely (Figure 2A). Our date suggested that mPG-1 may serve as a ligand of IGF1R, and selectively activate the CREB, but not other classical transcription factors in this growth factor family (Adiseshaiah et al., 2008), the EGFR pathway may be involved in the regulation as well.

**Figure 3:**
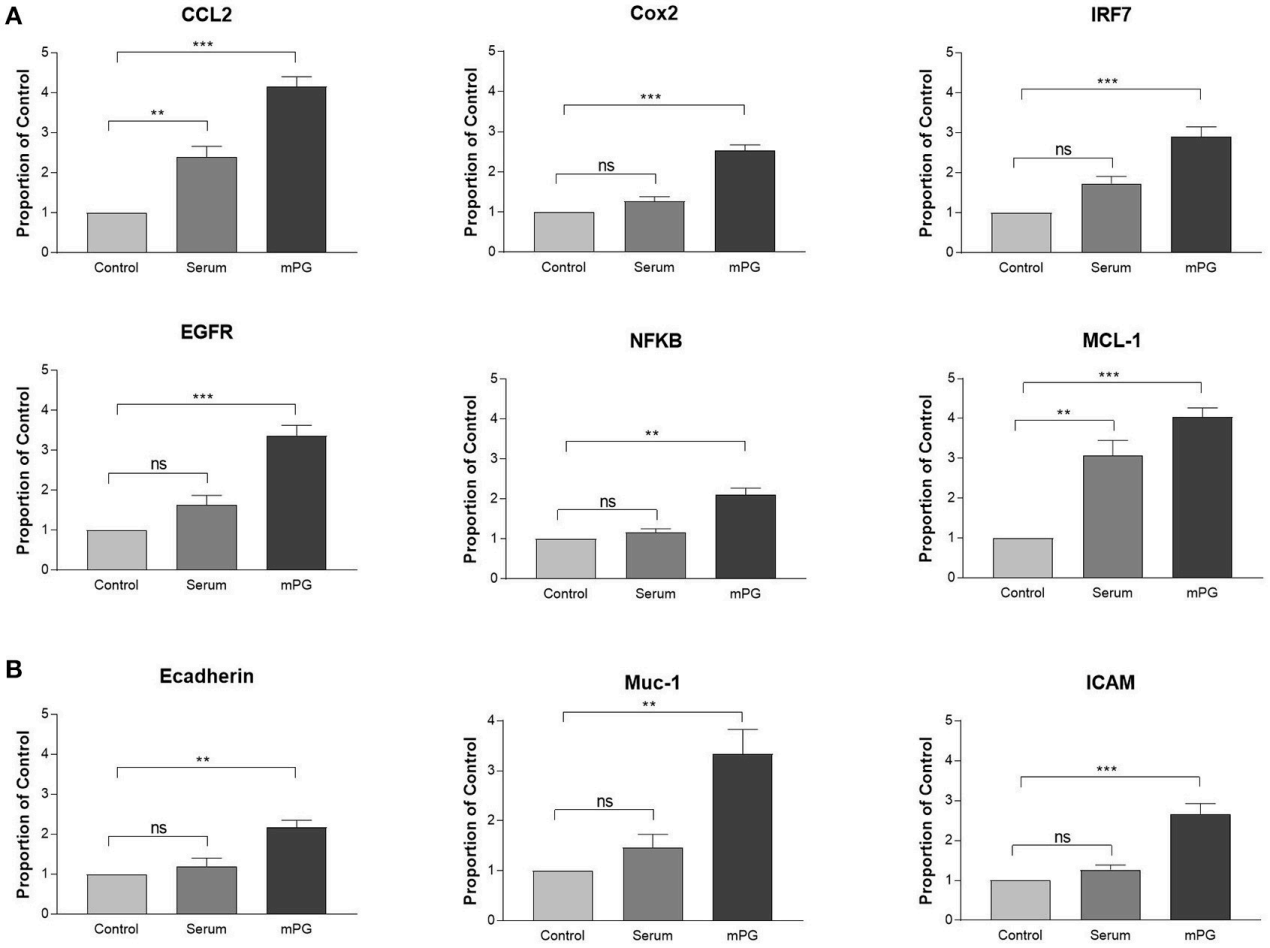
mPG-1 increased expression of pro-inflammatory and migration markers. **(A)** IPEC-J2 cells were treated with mPG-1 (9 μM) for 3 hours, RNA was isolated, and expression of pro-inflammatory cytokines and cell migration markers **(B)** was assessed using qRT-PCR. Bars represent the mean ± SEM of three experiments. ns, Not Significant, **p < 0.01 and ***p < 0.001 as calculated by a one-way ANOVA *post hoc* Tukey test.

It is well known that activation of IGF1R leads to enhanced expression of both inflammatory mediators and genes that are associated with cell migration (Li et al., 2009, 2017; Smith, 2010; Bilbao et al., 2014; Mancini et al., 2014). To investigate if mPG1 activation of IGF1R also influenced expression of inflammatory mediators and genes associated with migration, IPEC-J2 cells were cultured in the absence and presence of mPG-1. The expression of genes directly or indirectly involved in the immune-response; C-C Motif Chemokine Ligand 2 (CCL2), Cyclooxygenase 2 (COX2), Interferon Regulatory Factor 7 (IRF7), Nuclear factor-κB (NFKB) epidermal growth factor receptor (EGFR), and myeloid cell leukemia-1 (MCL-1), was analyzed using qRT-PCR (Figure 3A). In addition, we analyzed the expression of Ecadherin (Ecad), Mucin-1 (Muc-1) and intercellular adhesion molecule 1 (ICAM), are all known to be associated with cell migration (Kevil et al., 2004; Campbell and Casanova, 2015; Wang et al., 2015), and each were shown to have increased expression in response to mPG-1 (Figure 3B), consistent to our observation that cell migration was enhanced by the AMP.

**Figure 4:**
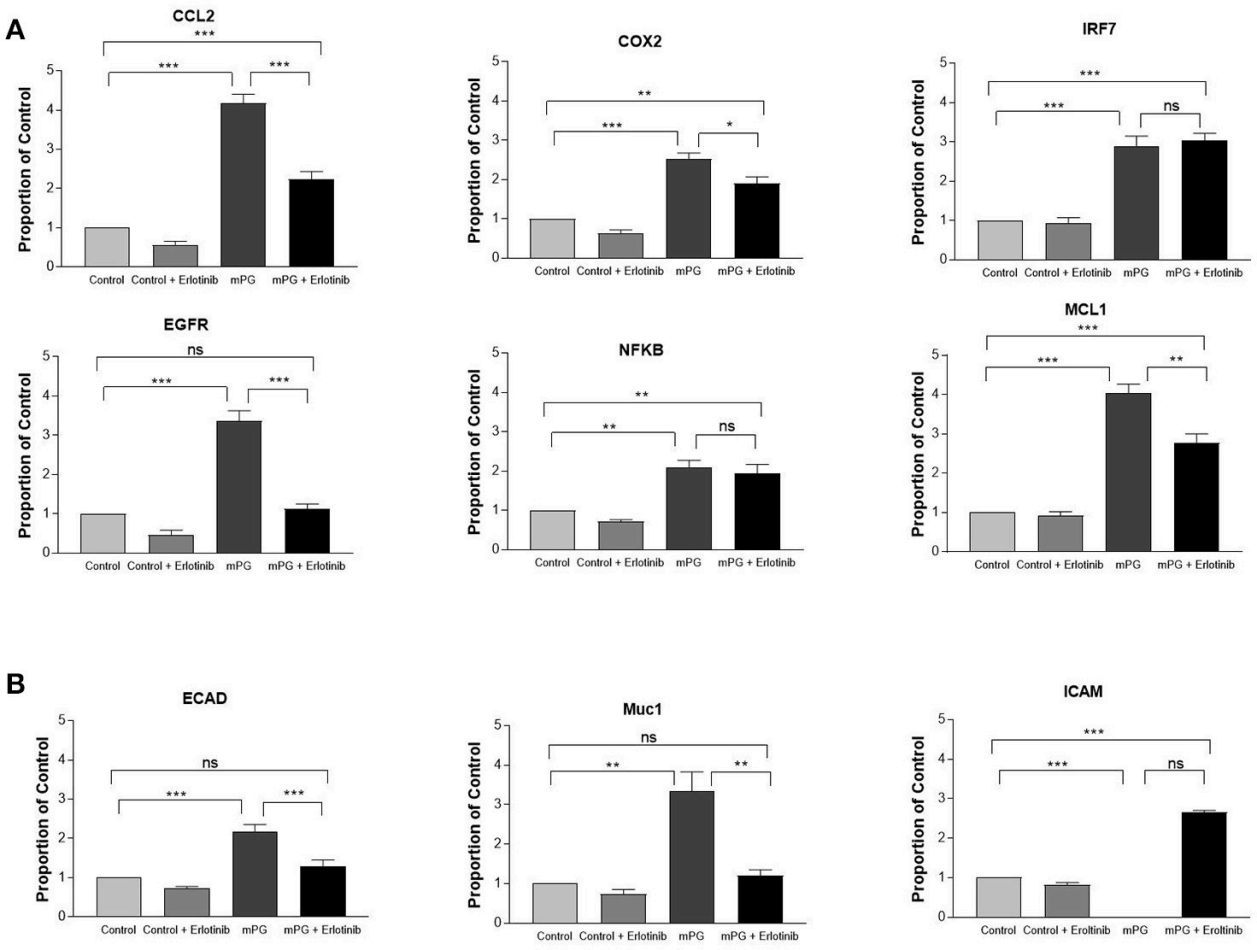
mPG-1 induced changes in gene expression are partially inhibited by erlotinib (E: EGFR inhibitor) **(A)** IPEC-J2 cells were treated with erlotinib (10 μM) for 30 min prior to the mPG-1 (9 μM) for 3 h, RNA was isolated, and expression of pro-inflammatory cytokines and cell migration markers **(B)** was assessed using qRT-PCR. Bars represent the mean ± SEM of three experiments. ns, Not Significant, *p < 0.05, **p < 0.01 and ***p < 0.001 as calculated by a one-way ANOVA *post hoc* Tukey test.

Activation of EGFR and IGF1R can both lead to activation of downstream pathways, ERK and AKT, and activation of either pathway may enhance the expression of both inflammatory mediators and migration-related genes (Desbois-Mouthon et al., 2009; Hanson et al., 2010; Schanzer et al., 2016). To further verify that mPG-1 activated IGF1R and to investigate if EGFR may also be activated by the antimicrobial peptide, we first pre-treated the cells with erlotinib, an inhibitor specific for EGFR (Wang et al., 2012), and assessed the expression of the same genes listed above. As shown in Figures 4A,B, with the exception of IRF7, NFKB and ICAM, mPG-1 induced expression of most of the cell migration and inflammatory mediator genes were partially suppressed (except for EGFR, Ecadherin where suppression was complete) by the EGFR inhibitor. This incomplete blockage of mPG-1 response by the inhibitor suggests that EGFR may be one of the pathways through which mPG-1 exerts its immunomodulatory and cell migration enhancing effects but is not the sole pathway activated.

**Figure 5:**
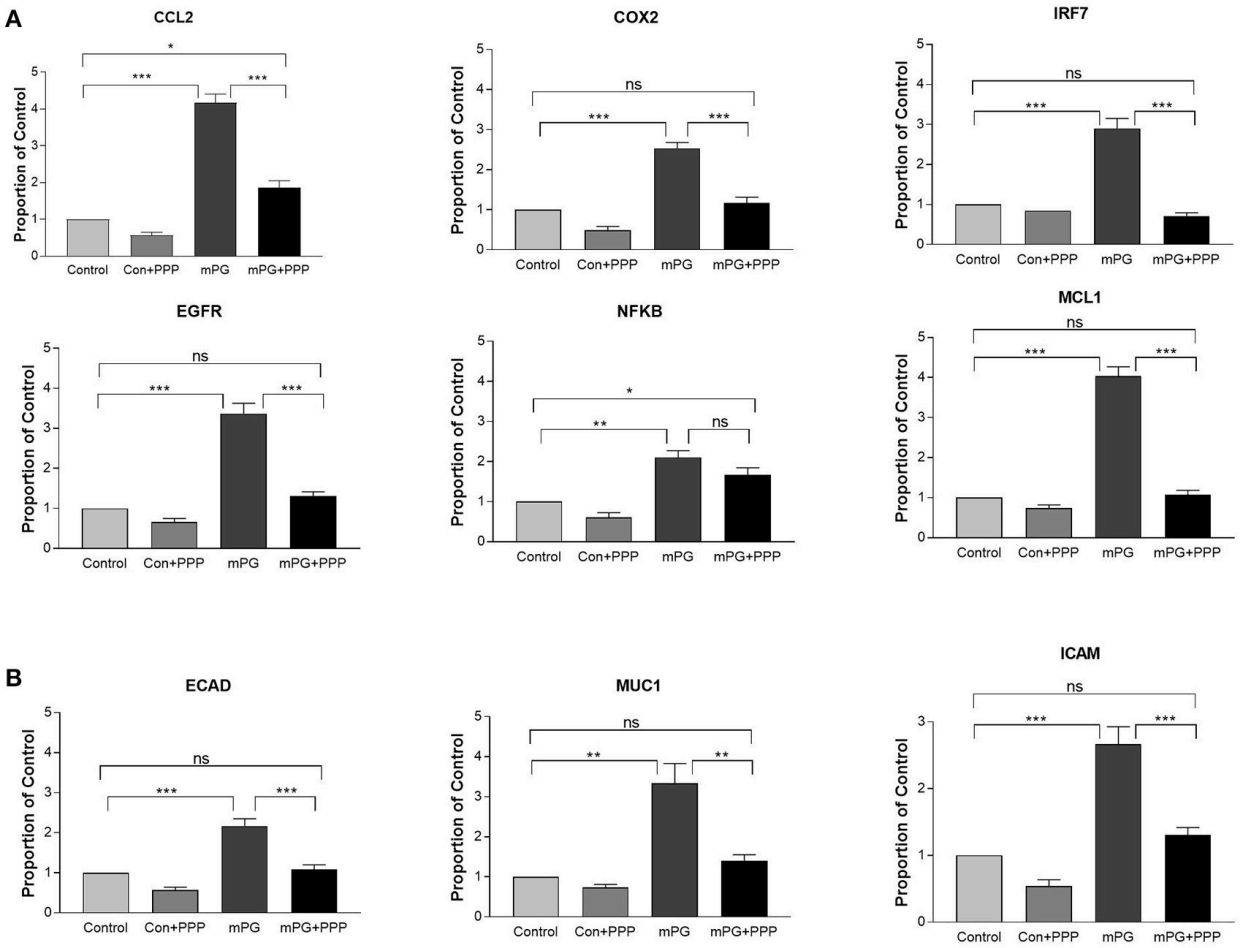
mPG-1 induced changes in gene expression are inhibited by picropodophyllin (PPP: an IGF1R inhibitor) **(A)** IPEC-J2 cells were treated with PPP (10 μM) prior to the mPG-1 (9 μM) for 3 h, RNA was isolated, and expression of pro-inflammatory cytokines and cell migration markers **(B)** was assessed using qRT-PCR. Bars represent the mean ± SEM of three experiments; ns, Not Significant, *p < 0.05, **p < 0.01 and ***p < 0.001 as calculated by a one-way ANOVA *post hoc* Tukey test.

When the intestine cell line was treated with IGF1R inhibitor, PPP prior to mPG-1 exposure, the increase in expression of most of the genes inflammation and cell migration associated genes observed with mPG-1 alone was completely reversed to the levels that are not significant to their respective controls, with the exception of only CCL2 and NFKB (Figure 5A). Additionally, the increase in expression of migration associated genes induced by mPG-1 was completely blocked by pre-treatment with PPP (Figure 5B). This suggests that mPG-1 exerts its immune-mediating and cell migratory effects, at least in part, or mainly through IGF1R in IPEC-J2 cells.

**Figure 6:**
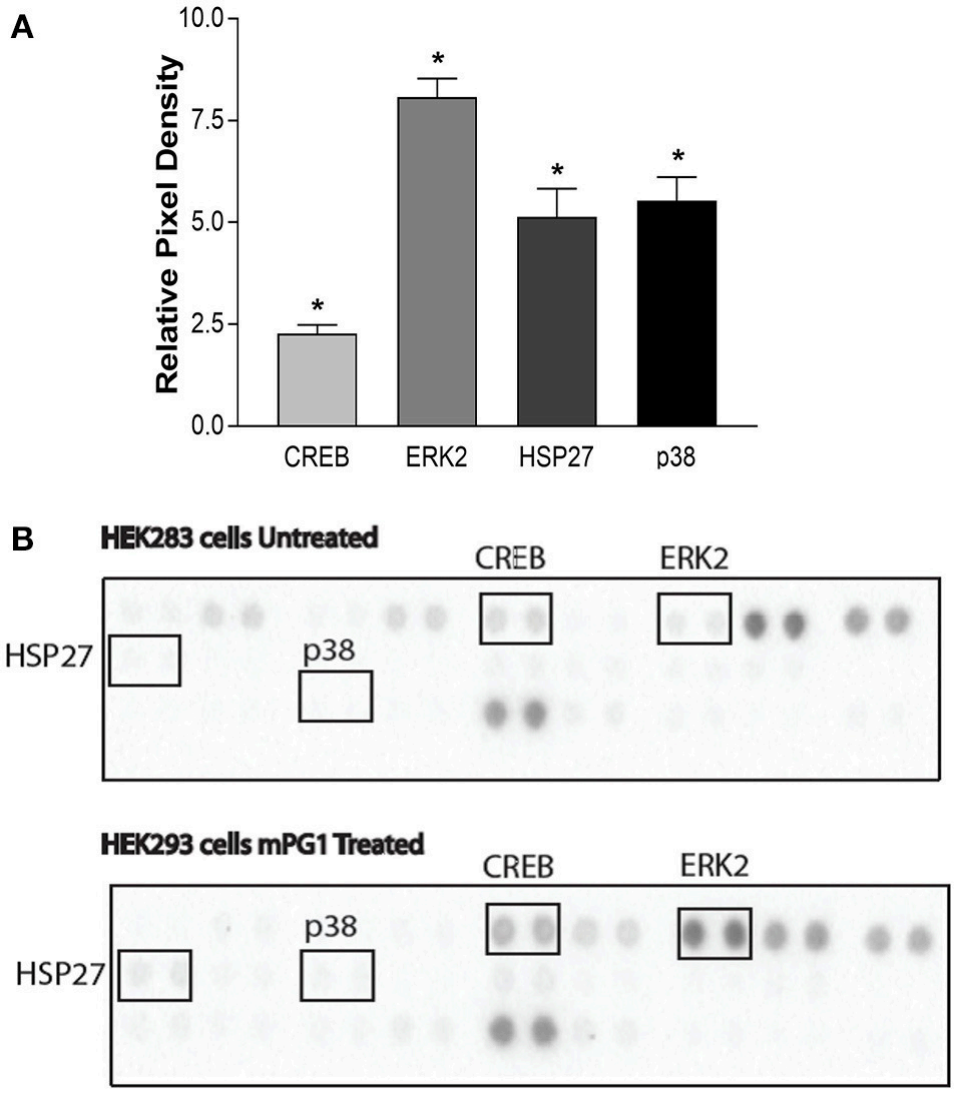
mPG-1 activates the ERK pathway. **(A,B)** HEK293 cells were serum starved for 24 h, either left untreated or were treated with mPG-1 (9 μM) for 15 min. The cells were then collected and run on the Proteome Profiler Human Phospho-MAPK Array to assess phosphorylation level of various proteins. **(A)** showing a representative blot for each group. **(B)** Quantification of the above blots relative to the control, untreated samples. Bars represent the mean ± SEM of three experiments. *p < 0.05 as calculated by a one was ANOVA *post hoc* Tukey test.

To further delineate the pathway(s) that mPG-1 activated, we used a well-defined array with commonly activated mitogen-activated protein kinases (MAPKs) related to IGF1R and EGFR. When IGF1R or EGFR is activated they form dimers and activate various downstream pathways, two of the major pathways being the ERK and AKT pathways (Sachdev and Yee, 2007). In order to investigate which of these pathways was activated by mPG-1, we used a proteome profiler to assess the relative phosphorylation status of 26 different MAPKs (Figure 6). It was found that the most highly activated factor was ERK2, along with other factors, such as CREB, HSP27 and p38, this is consistent with activation through IGF1R and EGFR pathways. While the AKT pathway was slightly downregulated (date not shown) suggesting that mPG-1 selectively activates the ERK pathway through IGF1R/EGFR without activating the AKT pathway.

## Discussion

Our current study confirms the intestinal cell migration stimulating effect of mPG-1, and also suggested that this AMP has an immune-mediating function. Further, we demonstrated that mPG-1 exerts these functions through activating IGF1R/EGFR. Our conclusion is supported by the following findings: (A) treatment with mPG-1 resulted in an increase in phosphorylation of ERK2; (B) mPG-1 induced expression of the cell migration associated- and/or immune-mediating genes and this was partially suppressed by the EGFR inhibitor, and almost completely reversed by the IGF1R inhibitor; (C) the transcription factor activity reporter assay revealed that mPG-1 activated CREB, a transcription factor downstream of IGF1R/EGFR, and this activation is partially (EGFR) and almost completely (IGF1R) reversed by the inhibitors of the growth factor receptors, respectively. Although the mechanism through which mPG-1 kills bacteria is well understood, to the best of our knowledge, this is the first study investigating the potential role of mPG-1 in immuno-mediation in intestinal cells, and the pathways that may be involved. Our findings suggest a role for mPG-1 not only in immune modulation but also in cellular migration, which could lead to potential implication in infection control and inflammation in the gastrointestinal tract.

We first sought to characterize any effect that mPG-1 may have on cell migration, as this is an important process for intestinal health and healing, which can limit the spread of infection (Heath, 1996). Our findings suggest that mPG-1 enhances migration in a dose dependent manner, we next studied how it exerted this effect on migration. Interestingly, there was no effect on proliferation with mPG-1 treatment (unpublished data). We assessed the expression of some migration marker genes and found that, when treated with mPG-1, their expression was enhanced. Two of the major receptors that are known to be involved in cell migration are the epidermal growth factor receptor (EGFR) and the insulin like growth factor 1 receptor (IGF1R) (Pollack, 2012; Li et al., 2017). To elucidate which pathway was responsible for this change in expression, we administered specific inhibitors for each receptor prior to mPG-1 treatment and found that while erlotinib (EGFR inhibitor) (Orcutt et al., 2011) partially blocked this effect, PPP (IGF1R inhibitor) (Vasilcanu et al., 2006) yielded a more complete, albeit still not 100%, reversal of the effect of mPG-1. This suggests that while IGF1R may not be the sole receptor responsible for the effects that mPG-1 has on migration, it appears to play a major role, and EGFR may play a more minor role in this mPG-1 action.

It is important to note that while we have shown that mPG-1 activates the IGF1R pathway, it is not clear whether this activation occurs through direct or indirect binding of mPG-1. It is possible that mPG-1 acts as a ligand and binds directly to the receptor, however we have no evidence for or against this. It is also possible that mPG-1 mediates the activity of an intermediate molecule that then results in receptor activation, a potential molecule could be Tripartite Motif Containing 29 (TRIM29) (Sun, 2017). TRIM29 binds nucleic acids and acts as a transcriptional regulatory factor and it has been shown to negatively regulate the innate immune response and is highly expressed in intestinal epithelial cells (Xing et al., 2017, 2018). It is possible that mPG-1 inhibits TRIM29 thereby lifting its negative regulatory effects on the innate immune response and increasing the activity of immune related pathways such as IGF1R.

**Figure 7:**
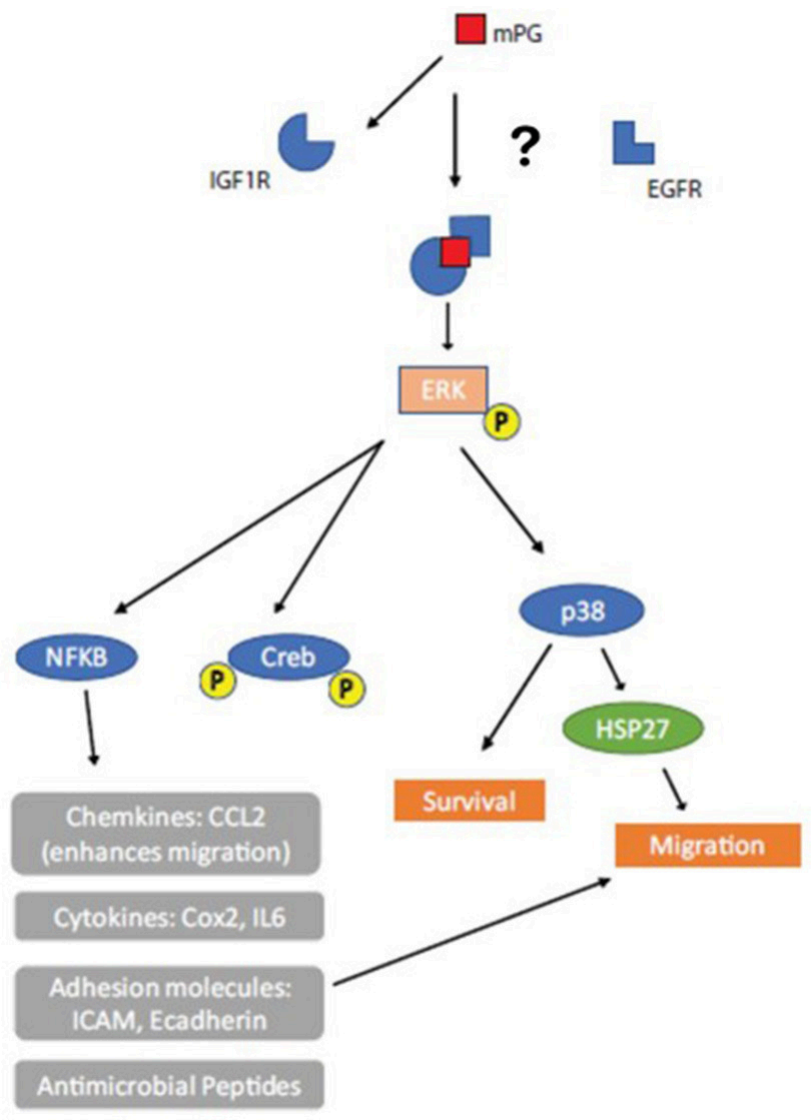
Working Model. mPG-1 activates IGF1R causing it to form homodimers and/or heterodimers (with EGFR). This activation causes downstream effects including ERK phosphorylation which promotes an increase in expression of pro-inflammatory cytokines and cell migration.

Our findings on the potential pathway involved in the effects mPG-1 has on the expression of genes involved in inflammation revealed a similar response to those from the cell migration associate genes, in that the EGFR inhibitor provided a partial block, while IGF1R provided a more complete block of the increase in expression of cytokines. We investigated the activity of some integral transcription factors using a transcription factor activity reporter system of different sub-pathways, including CREB, c-Jun and ELK1. It was found that only the activity of CREB was enhanced by mPG-1 and that this effect was again negated by a pre-treatment with the inhibitors of EGFR (partially) and IGF1R. CREB is involved in integral biological processes including differentiation, migration, and cellular adaptation. It is also believed to be important for learning and memory and contribute to hormonal control of metabolic processes (Shaywitz and Greenberg, 1999). The lack of increased activation of c-Jun and ELK1 observed in the study suggests that not all of the major downstream pathways of IGF1R/EGFR were activated by mPG-1 (Peruzzi et al., 1999). Our finding, from the proteome phosphorylation profile array, that mPG-1 induced phosphorylation of the ERK pathway without activation (and even slight depression) of the AKT pathway, is consistent with this notion. Both IGF1R and EGFR are activated by ligands which causes them to form homo- and hetero-dimers in order to activate downstream pathways (Maruyama, 2014). It has also been shown that IGF1R can form heterodimers with human epidermal growth factor receptor 2 (HER2) under certain circumstances, HER2 is in the same receptor family as EGFR (Li et al., 2009). We therefore postulate that mPG-1 may cause IGF1R activation which leads to homodimerization and activation of downstream targets; but also, that some of the effects may be due IGF1R forming heterodimers with EGFR. Taking into account the data presented, we proposed a working model for potential pathways for mPG-1 cell function and gene expression regulation in IPEC-J2 cell (Figure 7). It is important to note that this pathway is not necessarily the only pathway through which mPG-1 exerts its effects and that the proposed method of activation is a hypothesis as direct binding and the formation of heterodimers has not been proven.

Previous studies have shown that the human cathelicidin AMP LL-37 binds to and activates IGF1R as a partial agonist, activating the ERK pathway without AKT activation (Girnita et al., 2012). Our study suggests that mPG-1 may act through similar pathways as LL-37 despite their differences in structure. In this LL-37 study, it was found that treatment with LL-37 enhanced cellular migration without affecting proliferation, this mirrors what we found in our mPG-1 study (Girnita et al., 2012). These similarities in mechanisms and effects suggest that some important AMPs may work through similar mechanisms despite coming from different species and cell types. While the structure of AMPs can vary, their mechanisms may be evolutionarily conserved due to their importance in defense against pathogens.

A previous study investigated the mechanism through which mPG-1 regulates mast cells degranulation, and thus plays a role in immune modulation. It was found that mPG-1 bound to the membrane localized receptor MrgX2 (Gupta et al., 2015). However, MrgX2 is mast cell specific, and therefore it is likely that mPG-1 exerts its immunomodulatory effects through additional receptors expressed in other cell types (Gupta et al., 2015). Our finding on the involvement of IGF1R and EGFR in mPG-1 adds additional insights onto the working mechanism of this AMP in non-immune cells.

AMPs and their role in antimicrobial activity, tissue repair and immune-protection represent a potential treatment for infection particularly in disorders involving the digestive system as the spread of pathogens is always a threat (Cunliffe and Mahida, 2004). Inflammatory bowel disease (IBD) is an umbrella term used to describe disorders that involve chronic inflammation of the gastrointestinal tract (GI) and these disorders can lead to decreased epithelial barrier function (Hanauer, 2006). This is an important function as it aids in preventing any pathogens from getting through the GI tract into the blood stream. In clinical IBD studies, mucosal healing has emerged as a main treatment goal in IBD as it is a reliable indicator of successful healing of gut inflammation (Papi et al., 2013). Gut tissue repair is an orchestrated process requiring the integration of events involving inflammation, angiogenesis and tissue regeneration (Eming et al., 2014). In IBD patients, the colonic expression of cathelicidin is increased in ulcerative colitis patients compared to the non-inflamed control mucosa, suggesting this peptide may have a role in modulating GI tract inflammation (Nuding et al., 2007). Given the data we presented in this study, suggesting that mPG-1 enhances the immune response and cellular migration and thus has tissue repair potential, it is possible that it would enhance healing of the GI tract in patients with IBD.

## Author contributions

All authors listed have made a substantial, direct and intellectual contribution to the work, and approved it for publication.

### Conflict of interest statement

The authors declare that the research was conducted in the absence of any commercial or financial relationships that could be construed as a potential conflict of interest.

## Footnotes

^1^Drexler HG et al. (eds): DSMZ Catalog of Human and Animal Cell Lines, www.dsmz.de

^2^Note the alamar blue assay was performed in the presence and absence of serum and the viability trends were the same.
