# Supplementary figures and images for: Protegrin 1 Enhances Innate Cellular Defense via the Insulin-Like Growth Factor 1 Receptor Pathway
Source: Front Cell Infect Microbiol. 2018 Sep 28;8:331. doi: 10.3389/fcimb.2018.00331 (PMC6173103; doi:10.3389/fcimb.2018.00331)

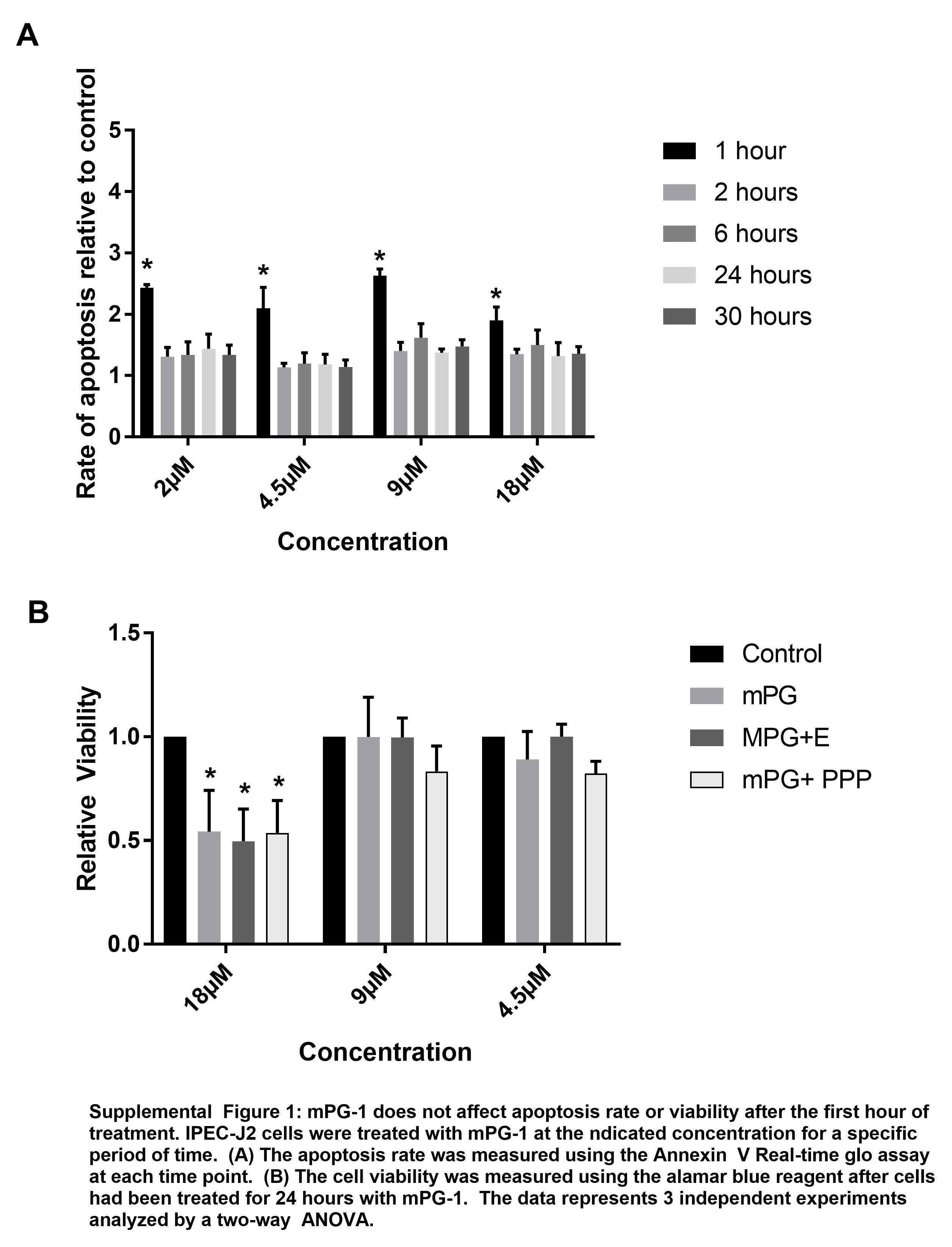

Supplement: Supplementary file 1 [file Image_1.jpg]
